# Antiviral Screen against Canine Distemper Virus-Induced Membrane Fusion Activity

**DOI:** 10.3390/v13010128

**Published:** 2021-01-18

**Authors:** Neeta Shrestha, Flavio M. Gall, Jonathan Vesin, Marc Chambon, Gerardo Turcatti, Dimitrios Fotiadis, Rainer Riedl, Philippe Plattet

**Affiliations:** 1 Division of Experimental and Clinical Research, Vetsuisse Faculty, University of Bern, CH-3012 Bern, Switzerland; neeta.shrestha@vetsuisse.unibe.ch; 2 Institute of Chemistry and Biotechnology, Center for Organic and Medicinal Chemistry, Zurich University of Applied Sciences (ZHAW), CH-8820 Wädenswil, Switzerland; galf@zhaw.ch (F.M.G.); rira@zhaw.ch (R.R.); 3 Biomolecular Screening Facility, Ecole Polytechnique Fédérale de Lausanne (EPFL), CH-1015 Lausanne, Switzerland; jonathan.vesin@epfl.ch (J.V.); marc.chambon@epfl.ch (M.C.); gerardo.turcatti@epfl.ch (G.T.); 4 Institute of Biochemistry and Molecular Medicine, and Swiss National Centre of Competence in Research (NCCR) TransCure, University of Bern, CH-3012 Bern, Switzerland; dimitrios.fotiadis@ibmm.unibe.ch

**Keywords:** CDV, cell-based fusion assay, envelope glycoproteins, host cell receptor, inhibitor discovery

## Abstract

Canine distemper virus (CDV), a close relative of the human pathogen measles virus (MeV), is an enveloped, negative sense RNA virus that belongs to the genus *Morbillivirus* and causes severe diseases in dogs and other carnivores. Although the vaccination is available as a preventive measure against the disease, the occasional vaccination failure highlights the importance of therapeutic alternatives such as antivirals against CDV. The morbilliviral cell entry system relies on two interacting envelope glycoproteins: the attachment (H) and fusion (F) proteins. Here, to potentially discover novel entry inhibitors targeting CDV H, F and/or the cognate receptor: signaling lymphocyte activation molecule (SLAM) proteins, we designed a quantitative cell-based fusion assay that matched high-throughput screening (HTS) settings. By screening two libraries of small molecule compounds, we successfully identified two membrane fusion inhibitors (F2736-3056 and F2261-0043). Although both inhibitors exhibited similarities in structure and potency with the small molecule compound 3G (an AS-48 class morbilliviral F-protein inhibitor), F2736-3056 displayed improved efficacy in blocking fusion activity when a 3G-escape variant was employed. Altogether, we present a cell-based fusion assay that can be utilized not only to discover antiviral agents against CDV but also to dissect the mechanism of morbilliviral-mediated cell-binding and cell-to-cell fusion activity.

## 1. Introduction

Canine distemper is a highly infectious disease caused by the canine distemper virus (CDV), a member of the family *Paramyxoviridae* and closely related to the measles virus (MeV) human pathogen. CDV is a negative sense single stranded RNA virus that is present worldwide and has a wide host range within the order Carnivora. While domesticated dogs are common hosts, CDV also infects various wild species within various families, namely, *Canidae*, *Hyaenidae*, *Phocidae*, *Felidae*, *Procyonidae*, *Mustelidae*, *Ursidae*, *and Viverridae*, thereby threatening the lives of many endangered animals [1,2,3,4,5].

Primarily, CDV infection in dogs is transmitted by inhalation of infectious aerosol droplets but transmission by direct contact with body fluids or fomites is also possible [6]. Post inhalation, CDV establishes the infection by binding a receptor: signaling lymphocyte activation molecule family member 1 (SLAM/F1, or CD150), expressed by dendritic cells (DCs), subsets of thymocytes, macrophages, and T- and B-lymphocytes [7,8]. This facilitates the systemic dissemination whereby the virus interacts with a second receptor: nectin cell adhesion molecule 4 (nectin-4), expressed at the basolateral surface of epithelial cells [8], thereby infecting various tissues like fibroblasts, keratinocytes, gastrointestinal mucosa and respiratory tract. Infection in the airway epithelium results in the virus assembly and the release of virions into the airway lumen of the infected lung [9], whereby the virus can be transmitted to a new host. CDV can also invade the central nervous system (CNS) and cause either acute polioencephalitis or demyelinating leukoencephalitis depending on the strain of CDV [10,11].

CDV enters the host cell by engaging two glycoproteins present on its surface, the attachment (H) protein and the fusion (F) protein, which fuses the lipid bilayer of the viral envelope and the target cell plasma membrane. Upon receptor binding, it is speculated that both surface glycoproteins undergo series of specific structural arrangements, which in turn results in merging of the host and viral lipid bilayers and formation of a pore. While H and F can mediate virus-to-cell fusion, they can also cause cell-to-cell fusion, which eventually results in syncytium formation. Even in the absence of the virus, sole expression of both surface glycoproteins in cell culture systems results in syncytium formation. Such a phenotype could also be employed to design a targeted bioassay.

Although vaccination is available and effective in dogs, problems such as vaccine-failure and even vaccine-based fatal outcome in highly susceptible animals have been reported [12,13,14]. Various approaches to identify antiviral compounds against CDV have been presented earlier [15,16,17]. Moreover, two fusion inhibitor families were reported where one group was peptide based inhibitors with FIP (fusion inhibitory peptide, Z-D-Phe-L-Phe-Gly-OH) as the most prominent representative and the other group was based on the 2-phenylacetanilide scaffold with AS-48 and 3G as prominent examples [18,19,20]. However, the moderate efficacy (low-to-sub micromolar range of 50% inhibitory concentrations) hindered further development of the respective molecules [21,22]. In addition, high-throughput screening (HTS) of small molecule compounds using reporter protein-expressing recombinant live-viruses yielded a promising pan-morbillivirus inhibitor (i.e., ERDRP-0819), which likely targets the large (L) protein of the polymerase complex [23]. Although very powerful, a potential drawback of using recombinant live-viruses with defined output methods for screenings, is the discovery of molecules “trapped” to certain viral antigens involved in potentially “dominant” viral functions (e.g., F-protein (cell entry) or L-protein (replication)), thereby hampering the chances to identify inhibitors targeting other desired viral components [24]. Problems as such can be eventually mitigated by designing rather targeted bioassays that mimic designated stages of viral life cycle.

In this study, we developed a quantitative cell-based fusion assay matching with HTS settings. The assay relied on two glycoproteins (H and F), the cognate receptor SLAM and the highly sensitive nanoluciferase reporter system. Upon the screen of two libraries of about 14,000 small molecule compounds, we identified two potent fusion inhibitors (F2736-3056 and F2261-0043). Although structural analysis revealed similarities of both compounds with the AS-48 class molecules, F2736-3056 exhibited better efficacy in blocking fusion activity mediated by a hyperfusogenic “3G-escape” CDV F-protein variant. Thus, in addition to discover novel CDV entry inhibitors, our assay also spotlights the attractive potential to identify molecular tools enabling dissecting the mechanisms of morbilliviral-induced cell-entry and spread.

## 2. Materials and Methods

### 2.1. Cell Culture

Vero cells (ATCC CCL-81), Vero cells stably expressing canine SLAM (Vero-cSLAM, kindly provided by Yusuke Yanagi, Kyushu University, Fukuoka, Japan), Vero-cSLAM-Green Fluorescent Protein (GFP)/LgBiT, Vero-Fs-sH-Red Fluorescent Protein(RFP)/HiBiT and HEK-293T cells (TCC CRL-11268) were used in this study and maintained in Dulbecco’s modified eagle’s medium (Gibco, Invitrogen, Carlsbad, CA, USA) containing 10% fetal calf serum (FCS; BioSwissTech, Schaffhausen, Switzerland) and penicillin-streptomycin (pen-strep) at 37 °C and 5% CO_2_ (excluding the compound screening experiment where Vero and Vero-cSLAM cells were cultured in absence of pen-strep). The NanoLuc^®^ Binary Technology (NanoBiT; Promega, Madison, WI, USA) is a structural complementation reporter system composed of a Large BiT (LgBiT; 18 kDa) subunit and a small complimentary peptide (e.g., HiBiT; high affinity to LgBiT). Vero-Fs-sH-RFP/HiBiT cells were cultured in presence of 10 µM of Asunaprevir.

### 2.2. Stable Transfection

CDV surface glycoprotein (F and H; Onderstepoort strain)-expressing genes, fused (N-terminal: H protein, C-terminal: F protein) to the Small Molecule Assisted Shut-off (SMASh) tag referred to as Fs and sH, respectively [25], were synthetically generated by Eurofin Genomics (Ebersberg, Germany). CDV surface glycoproteins as well as the GFP-LgBiT and RFP-HiBiT [26] constructs were stably expressed in either Vero or Vero-cSLAM cells using the lentivirus technology, as previously described [27]. Vero-cSLAM-GFP/LgBiT and Vero-Fs-sH-RFP/HiBiT cells were cloned by limiting dilution technique. Vero-cSLAM were transfected with TransIT-LT1 (Mirus) by following the manufacturer’s instructions.

### 2.3. Compound Library

Two different commercially available libraries, (A) protein–protein interaction (PPI) library of around 5400 compounds and (B) chemical diverse collection of around 8000 compounds, were tested. Molecules were selected by the Biomolecular Screening Facility (BSF, EPFL, Lausanne, Switzerland) and were purchased from three different suppliers: Enamine, Life chemicals and ChemDiv. The entire process of screening was done at the biomolecular screening facility (BSF): a multidisciplinary technological platform at the EPFL for performing the high-throughput screening in life sciences-related projects.

### 2.4. Nanoluciferase (nLuc)-Based Assay for High-Throughput Screening

Using an ECHO550 liquid handler (Beckman Coulter), 40 nL of the stock compound (10 mM in 100% DMSO) was added to the Greiner bio-one 384-wells plate (40 μL final volume, final concentration 10 μM). Then, 100 μL (containing about 17,000 cells) of each cell suspension (Vero/cSLAM-GFP/LgBiT and Vero-Fs-sH-RFP/HiBiT) was seeded to the wells using a Multidrop Combi Reagent Dispenser (Thermo Scientific, Waltham, MA, USA) and incubated at 37 °C for 24 h. After the incubation, corresponding substrate (Nano-Glo^®^ Live Cell Assay System, Promega) was added to the plate using Biotek Microflo Select Dispenser (Agilent) and the total amount of luminescence was measured by Synergy NEO HTS Multi-Mode Microplate Reader (Biotek). Afterwards, all the statistical analysis was done by the BSF using the internal Laboratory Information Management System (LIMS).

### 2.5. CDV Inhibition Assay

Using a reverse genetic system for CDV (Onderstepoort (OP) strain; manuscript in preparation), the nLucP gene (encoding for the nanoluciferase fused to the “Pest” degradation motif; Promega) was inserted as an additional transcription unit in between the P and M genes of CDV-OP, which additionally contained the mNeonGreen gene (Neon) N-terminally attached to the CDV-OP N gene via a P2A motif (manuscript in preparation). Both viruses were successfully rescued and referred to as OP^neon^ and OP^neon/nLucP^, respectively. OP^neon/nLucP^ was used in all viral inhibition assays. A wild-type CDV virus (strain A75/17) was rescued, as previously described [28]. The latter recombinant virus harbors the Neon-P2A-nLucP cassette as an additional transcription unit inserted in between the P and M genes, and referred to as A75/17^neon/nLuP^. Desired inhibitors, dissolved in DMSO, were added in 96 wells plate (Greiner bio-one) starting at 100 nM with increasing concentration of half a log up to 100 µM. OP^neon/nLucP^ was then added to the plates at a multiplicity of infection (MOI) of 0.04 and incubated at 4 °C for 1 h. Mixtures were then added to Vero-cSLAM cells pre-seeded in a separate 96 wells plate and incubated at 37 °C for 24 h. The effect of the inhibitors was evaluated by measuring the amount of luminescence emitted by OP^neon/nLucP^-infected Vero-cSLAM cells using a multiplate reader (Cytation 5, Biotek, Winooski, VT, USA). After the measurement, all statistical analysis was carried out using the GraphPad Prism 8 package.

### 2.6. Viability Assay

In order to investigate the viability of cells in presence of hit compound, desired compounds were added in 96 wells plate (Greiner bio-one), similar to inhibition assay, starting at 100 nM with increasing concentration of half a log up to 100 µM. MT cell viability substrate and *NanoLuc*^®^ enzyme (RealTime-Glo™ MT Cell Viability Assay; Promega) were added to the Vero-cSLAM cell suspension, according to the manufacturer’s instruction. After, 15,000 Vero-cSLAM cells were added per well in the plate containing compounds and incubated at 37 °C for 24 h. The total amount of luminescence was measured using a multiplate reader (Cytation 5, Biotek). After the measurement, all statistical analysis was carried out using the GraphPad Prism 8 package.

### 2.7. In Silico Studies—Molecular Docking with the F-Protein

The F-protein was prepared for the docking studies using the molecular operating environment (MOE) via the QuickPrep option applying the default values for temperature 300 K, pH 7 and salt 0.1. The ligands F2261-0043 and F2736-3056 were imported and 3D coordinates for the ligands were generated directly in MOE using energy minimize with a root mean square deviation (RMSD) gradient of 0.1. The Amber10: EHT force field was applied for the minimizing and docking experiments. We used the “triangle matcher” as placement methods with “London dG” scoring and the “rigid receptor” mode for the refinement with the “GBVI/WSA dG” scoring. Water molecules were not included in the calculation.

## 3. Results

### 3.1. Establishment of a Quantitative Cell-Based Fusion Assay

**Figure 1:**
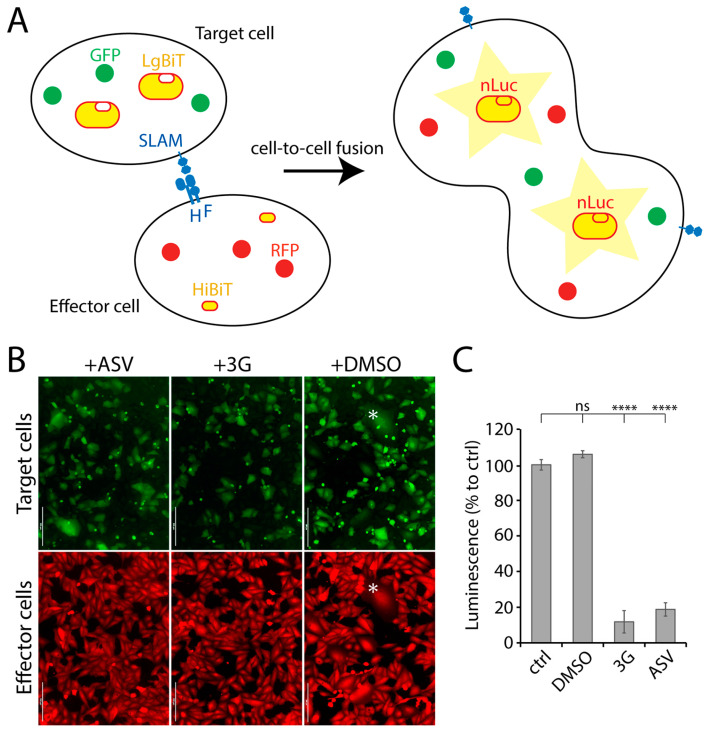
NanoLuc luciferase (nLuc)-based cell-to-cell fusion assay. (**A**) Schematic illustration of the cell-to-cell fusion assay. (**B**) Fluorescence micrographs of target (green) and effector (red) cells. Effector and target cells were mixed either in presence or absence of either 3G or Asunaprevir (ASV). The asterisk highlights syncytia expressing both the red and green fluorescent proteins. (**C**) Measurement of membrane fusion between effector and the target cells either in presence or absence of either ASV or 3G by recording luminescence emission. Each column represents replicate values from 3 independent experiments. Asterisks indicate statistical significance between the control and each group, as determined using one way ANOVA with Dunnett’s test (**** *p* < 0.0001). ns: not significant. Scale bars: 200 μm.

Provided that previous screens involving reporter protein-expressing recombinant live-viruses resulted in the discovery of anti-F or anti-L inhibitors [24], we investigated the possibility to increase the chances to identify novel antivirals by designing a more viral protein-targeted bioassay. We therefore established a quantitative cell-based fusion assay (potentially targeting viral cell entry and spread), which relied on the stable expression of the H, F and SLAM glycoproteins. Two cell lines were generated, namely effector and target cells. While effector cells (Vero) expressed CDV F- and H-proteins (Onderstepoort strain), target cells expressed the receptor only (Vero-cSLAM). Next, the split nanoluciferase (nLuc) system (Promega) was employed to generate a quantitative assay. Indeed, each part of split nLuc (HiBiT and LgBiT) were introduced into effector and target cells, respectively (Figure 1A). Moreover, in order to rationally control for CDV F and H expression, both glycoproteins’ cytosolic tails were fused to a tag, which allowed for protein expression-control (small molecule-assisted shutoff (SMASh) technology [25]). Briefly, by treating protein-expressing cells with the small molecule Asunaprevir (ASV), target protein-degradation is induced [25]. In summary, the assay relied on mixing effector and target cells together in absence of ASV, and 24 h later, to record the nanoluciferase (nLuc) activity. Thus, nLuc activity relied on productive F/H/SLAM interactions. Such glycoprotein engagements ultimately led to membrane fusion promotion (Figure 1B), which in turn triggered cytosolic content-mixing and, finally, nLuc reconstruction.

Addition of the nLuc substrate to such a system resulted in emission of luminescence, which indirectly corresponded to the total amount of membrane fusion. When membrane fusion activity was intervened using either 3G (a known fusion inhibitor; [22]) or ASV, luminescence signal was significantly diminished (Figure 1C), which verified our quantitative cell-based fusion assay as a very sensitive and convenient system to initiate screens for fusion inhibitors.

### 3.2. Screening of Small Molecules Using a Quantitative Cell-Based Fusion Assay

Firstly, the newly developed quantitative cell-based fusion assay was validated for its coherence with the high throughput screening (HTS) settings. In order to do that, reference controls (3G; positive control and DMSO; negative control) were assayed first. Using the control data, respective means and standard deviations (SD) were calculated, which in turn were utilized to calculate Z’ score. Z’ score is a characteristic parameter that evaluates the quality of an assay [29]. In our case, the measured Z’ score was about 0.5, which qualified the reliability of the assay. Next, approximately 5400 compounds from the PPI library and 8000 compounds from the CDC library were screened at a concentration of 10 µM. Every compound was tested in duplicates. After the screening, the data was analyzed using LIMS software package. The criteria for “hit” declaration were (a) measured value of the compound is above “average + 3X SD” of value of negative control and (b) both duplicates are active.

**Figure 2:**
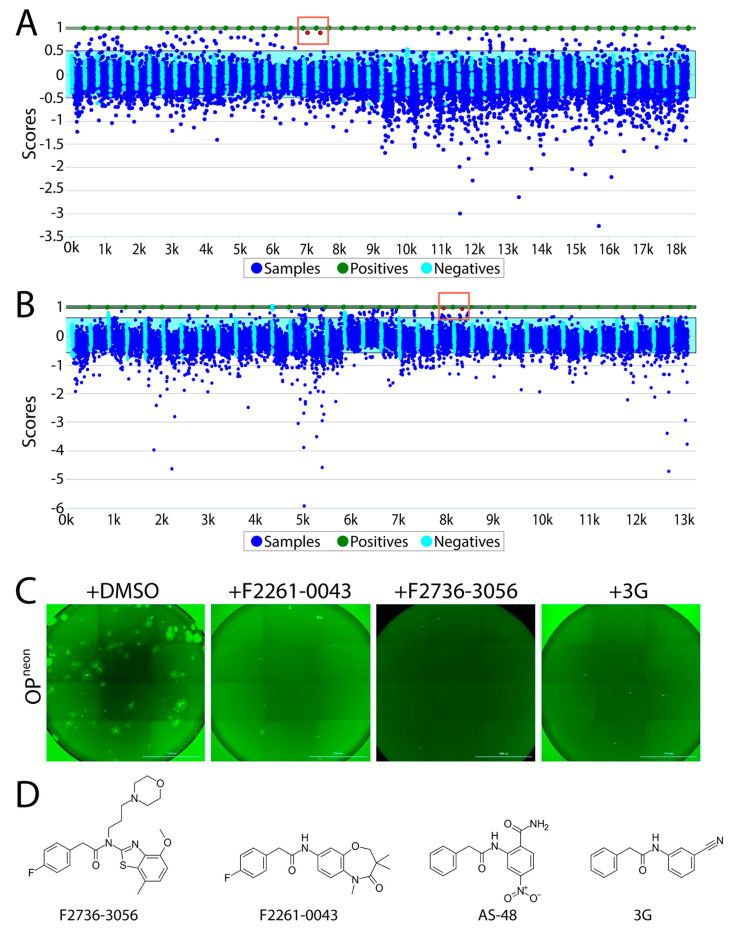
High-throughput screening of small molecules from the two independent libraries. (**A**) Laboratory Information Management System (LIMS) plot showing the result of small molecule screening from the CDC library. The positive control is normalized to have a score of 1 (equivalent to 100% inhibition) and the negative control is normalized to have a score of 0 (equivalent of 0% inhibition + 100% activity). While the Y axis displays the value of each data point relative to the controls, the X axis illustrates the samples screened (in duplicates). Green dots indicate the positive control (i.e., 3G); sky blue cyan dots represent the negative control (DMSO); dark blue dots show the compounds screened. Red dots in the red box indicate the selected top hit (in duplicates). While the light blue window represents the 3 standard deviations of the negative controls, the green window is the equivalent for the positive control. (**B**) LIMS plot showing the result of screening from the protein–protein interaction (PPI) library. Red dots in the red box indicate the selected top hit (in duplicates). (**C**) Infection inhibition analyses. Vero-cSLAM cells were infected with OP^neon/nLucP^ at a multiplicity of infection (MOI) of 0.04 either in absence or presence of either F2261-0043 or F2736-3056 (30 µM). (**D**) Structures of the compound F2736-3056, F2736-3056, AS-48 and 3G. Scale bars: 2000 μm.

Overall, using the abovementioned screening pipeline, we identified 60 compounds as “hits” (Figure 2A,B). Out of those compounds, F2736-3056 (identified from CDC library) and F2261-0043 (identified from PPI library) exhibited the highest inhibition profile in the fusion assay (Figure 2A,B). All confirmed hits were finally counterscreened (each small molecule at 10 µM) against a recombinant attenuated Onderstepoort (OP)-CDV that additionally expressed mNeonGreen (OP^neon^). Post 24 h of infection of Vero-cSLAM with OP^neon^, GFP expression was qualitatively analyzed. Out of 60 compounds, F2736-3056 and F2261-0043 successfully inhibited the GFP expression, which indirectly corresponded to CDV replication (Figure 2C). Upon comparison, the scaffold of both inhibitors resembled the membrane fusion inhibitor AS-48 class compounds (Figure 2D).

### 3.3. Potency of the Newly Discovered Fusion Inhibitors

**Figure 3:**
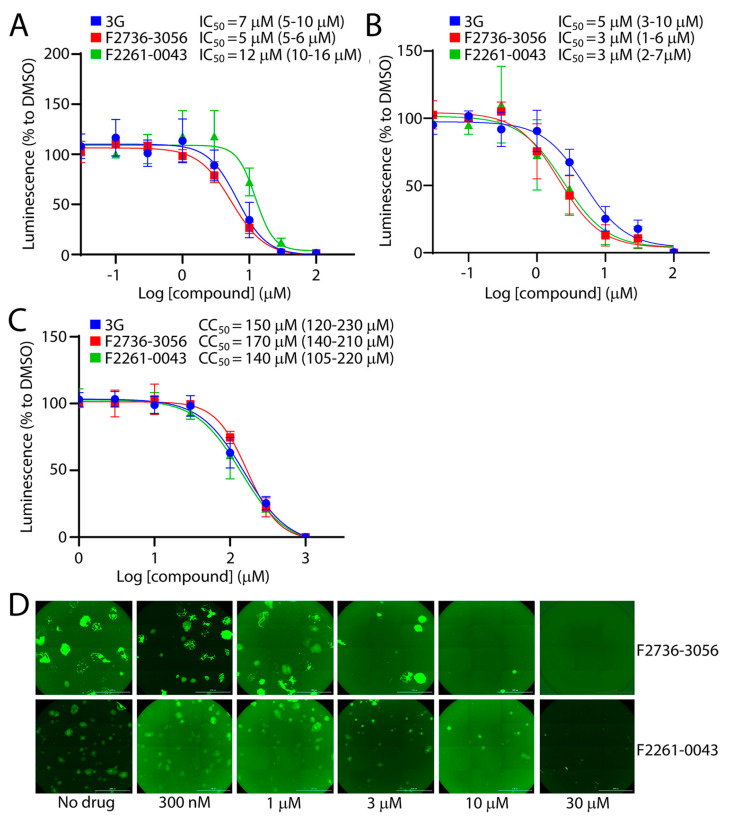
Infection inhibition efficiency of F2261-0043 and F2736-3056. (**A**) IC_50_ measurement of compounds against the attenuated OP-CDV strain. Vero-cSLAM cells were treated with increasing concentration of inhibitors (either F2261-0043, F2736-3056 or 3G) and infected with OP^neon/nLucP^ at an MOI of 0.04. Viral replication efficiency was determined by the measurement of luminescence produced by infected cells. (**B**) IC_50_ measurement of compounds against the wild type A75/17-CDV. Vero-cSLAM cells were treated with increasing concentration of inhibitors (either F2261-0043, F2736-3056 or 3G) and infected with A75/17^neon/nLucP^ at an MOI of 0.04. Viral replication efficiency was determined by the measurement of luminescence produced by infected cells. (**C**) Measurement of the cytotoxic effect of the inhibitors. Vero-cSLAM cells were treated with increasing concentration of the compound and assessed for the viability using real time Glo MT cell viability assay (Promega). (**D**) Assessment of the compounds’ inhibitory impact on virus-induced cytopathic effect (CPE). Microscopic images of cells infected with OP^neon^ in the presence of increasing concentration of the compounds (F2261-0043 and F2736-3056). IC_50_ and CC_50_ concentrations were calculated through four-parameter variable slope regression modeling using GraphPad Prism. All the 95% confidence intervals are shown in parentheses. The values for each concentration of the compound were normalized to DMSO-treated cells at identical concentration. Scale bars: 2000 μm.

Among the 60 tested compounds, F2736-3056 and F2261-0043 emerged as the most potent inhibitors of CDV infection. In order to accurately determine the efficiency of both small molecule compounds, we compared the bioactivity of these two inhibitors with 3G, which represents one of the most active derivatives of the AS-48 class compounds against CDV [22]. The compounds’ inhibition and cytotoxic impacts were assessed in Vero-cSLAM cells infected with OP^neon/nLucP^ (a recombinant virus expressing both the mNeonGreen and the nanoluciferase (nLucP) reporter proteins). While the 50% inhibition concentration (IC_50_) of compound F2736-3056 and F2261-0043 returned values of about 5 µM and 12 µM (Figure 3A), respectively, the 50% cytotoxic concentration (CC_50_) was about 170 µM and 140 µM (Figure 3C), respectively. Overall, while the selectivity index (SI) of compound F2736-3056 and F2261-0043 was about 34 and 12, the SI value for 3G was about 21. Indeed, 3G returned IC_50_ values of 7 µM and CC_50_ values of 150 µM (Figure 3A,C). Additionally, the inhibitory activity of the compounds was confirmed against a prototype wild-type CDV strains, namely A75/17, which is genetically distant from the OP-CDV strain. The compounds were also active against A75/17^neon/nLucP^, a recombinant wild-type virus expressing both, the mNeonGreen and nLucP reporter proteins (Figure 3B) (SI value for 3G: ~30; SI value for F2736-3056: ~56; SI value for F2261-0043: ~46).

In order to exclude the possibility that the compounds were directly inhibiting the nLuc enzymatic activity, we investigated whether the inhibitors were also affecting the cytopathic effect (CPE) induced by OP^neon/nLucP^, namely syncytia formation. Strikingly, a clear concentration-dependent decrease in number and size of the syncytia was observed (Figure 3D).

### 3.4. F2736-3056 Partly Preserved Fusion-Inhibition Activity against a 3G-Escape CDV F-Protein Variant

**Figure 4:**
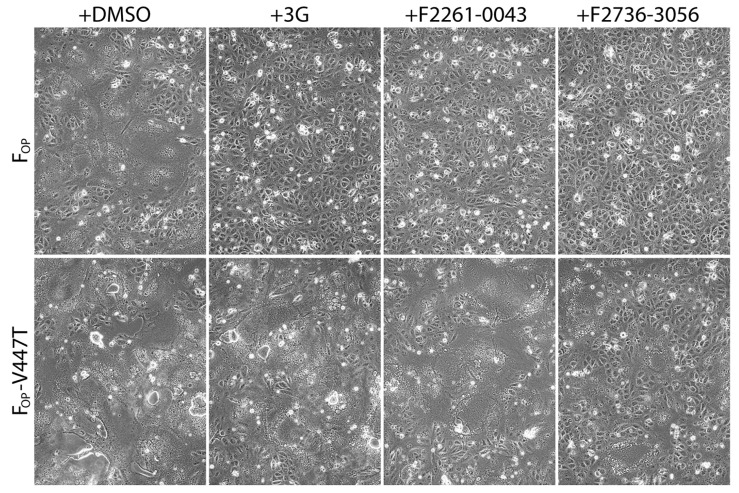
Enhanced inhibition efficiency of compound F2736-3056 against 3G-resistant F_OP_-mutant (V447T). Impact of the inhibitors was assessed using a previously described cell-to-cell fusion assay [30]. H_OP_ and either F_OP_ or F_OP_-V447T glycoproteins were co-expressed in Vero-cSLAM cells that were individually treated with DMSO (no compound), 3G, F2261-0043 and F2736-3056. Pictures of representative fields of view illustrating the extent of cell-to-cell fusion are shown for each condition.

Next, we tested the activity of F2736-3056 and F2261-0043 against a previously described 3G-escape, hyperfusogenic F-mutant (V447T; valine-to-threonine substitution at CDV F position 447) [27]. Of note, the 3G-escape profile of F-V447T precluded the synchronization of fusion activity necessary to run a quantitative cell-cell fusion assay. To tackle this limitation, we conducted a transient qualitative cell-cell fusion assay and recorded the extent of membrane fusion by light microscopy 24 h post-transfection. For this assay, H and F glycoproteins of Onderstepoort (OP) strain were used. While all three compounds efficiently inhibited cell-to-cell fusion induced by the standard F_OP_-protein (Figure 4), 3G and F2261-0043 lost their inhibitory activity in presence of F_OP_-V447T. In contrast, F2736-3056 preserved substantial inhibitory activity towards the hyperfusogenic F_OP_-V447T mutant, since membrane fusion remained partly inhibited (Figure 4).

### 3.5. In Silico Analysis Suggests a CDV F-Protein Hydrophobic Pocket as a Common Binding Site for Diverse Inhibitory Compounds

The binding mode of two fusion inhibitor families (FIP and AS-48) in MeV F was confirmed by X-ray crystallography [31]. The crystal structure of MeV F and the cryogenic electron microscopy (cryo-EM) structure of CDV F [32], both in the prefusion state, revealed high structural similarities.

The co-crystal structures of MeV F with AS-48 or FIP indicated that both binders dock into the similar hydrophobic region at the contact interface between two F-protomers, which locates at the junction of the head and stalk domains of the prefusion state. More specifically, the high-resolution structures revealed the key binding of a phenyl moiety of both inhibitors in a deep and narrow hydrophobic pocket (formed in proximity to Leu351 and Ile340 in MeV F [31]). In addition, a hydrophobic groove (formed in proximity to Pro241 and Pro339 in MeV F [31]), which extends from the deep pocket, is occupied by hydrophobic groups of AS-48 and FIP. Of note, is that the L-Phe side chain of FIP (absent in AS-48 and 3G) reaches an additional hydrophobic pocket (near Leu358 and Ala354 in MeV F [31]). This pocket is formed between two helices in the stalk domain.

**Figure 5:**
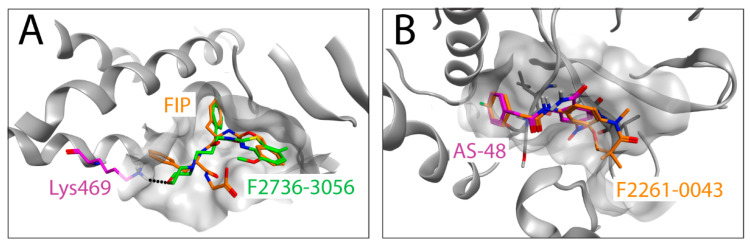
In silico docking of the identified compounds on an F-protein microdomain. The co-crystal structure (PDB-ID: 5YZC [30]) of the related measles virus fusion protein with the stabilizing inhibitor AS-48 was used to dock the newly identified hit compounds. (**A**) Docking pose of F2736-3056 (carbons in green) is shown in an overlay with FIP (carbons in orange). The lysine residue at position 469 of MeV F is shown in magenta, which corresponds to Lys581 of CDV F. Hydrogen bond is shown in black dots (**B**) Docking pose of F2261-0043 (carbons in orange) in an overlay with AS-48 (carbons in magenta) is shown.

Due to the lack of CDV F-structures in complex with ligands, we used the co-crystal structure of MeV F with AS-48 (PDB-ID: 5YZC) to investigate a potential docking mode of the two newly identified compounds. Interestingly, the docking models illustrate that F2736-3056 and F2261-0043 bind to the same hydrophobic region within the prefusion F-structure (Figure 5A,B). The 2-phenylacetanilide scaffold has been investigated in the past with AS-48 [19] and 3G [22] as prominent representatives. The structure activity relationship (SAR) of this scaffold showed a low tolerance for substitutions on the phenylacetic acid moiety. According to this SAR, the *para* fluoro substitution is the only tolerated substitution on this phenyl ring, which is consistent with the *para* fluoro substitutions of the newly identified hits F2261-0043 and F2736-3056. The seven-membered ring of F2261-0043 is placed in the shallow groove, which offers enough space for the additional fused ring (Figure 5B). The benzothiazole moiety of F2736-3056 was placed in the hydrophobic groove as the aniline moiety of AS-48 and the Z-group of FIP. The morpholine moiety is pointing towards the pocket occupied by the L-Phe side chain of FIP. The more polar morpholine is not entering into the hydrophobic pocket but the ether oxygen might form a hydrogen bond to the lysine residue at position 469 (the homologous amino acid in CDV F corresponds to the lysine residue at position 581) (Figure 5A). Collectively, our in silico approach with the two new hits indicated a potential overlap of the binding region with FIP and AS-48 in the previously experimentally determined co-crystal structures [31].

## 4. Discussion

Canine distemper virus is a negative sense ssRNA virus that is well-known to infect and cause fatal diseases in various animals. While initially recognized for causing distemper in domestic dogs, CDV is now well known for infecting wide range of hosts, including mostly but not confined to carnivore [1,2,3,4,5]. CDV is increasingly recognized as a major cause of decline in wild carnivore population [33], which inevitably raises concerns about the extinction threat of many endangered species [34]. In addition, lethal CDV outbreaks have been described in unprecedented species such as non-human primates demonstrating the remarkable ability of the pathogen to cross species barriers [35,36,37].

In 1960s, two Modified Live Virus (MLV) vaccines were developed as a control measure against CDV infection [38,39]. However, reports of post vaccinal canine distemper [40,41,42,43] led to the introduction of the recombinant CDV vaccines, which have been tested and shown to be safe to many susceptible species [44,45,46,47]. Albeit the suitability in many species, these recombinant vaccines have a limitation such as milder immunological response as compared to MLV vaccines [48]. As a therapy, there exists no specific antiviral drug against CDV infection in any species, including domestic dogs. Some promising candidates such as AS-48 class molecules (entry inhibitors) and pan-morbillivirus replication inhibitor were, however, recently reported [24]. Nevertheless, simultaneous discovering of more antivirals targeting different viral life cycle stages might mitigate issues such as generation of drug-resistant mutants.

In this study, we present a quantitative cell-based fusion assay, which enabled us to screen small molecule compounds potentially inhibiting CDV-mediated cell entry and spread. The split nature of the nanoluciferase indeed facilitated the indirect reading of the amount of cell-to-cell fusion (Figure 1C). The high sensitivity of the system and its optimization in 384-wells format offered an attractive framework to initiate HTS. Out of two independent libraries screened, we indeed identified two entry inhibitors, F2736-3056 and F2261-0043 (Figure 2), one from each library, which validated the power of the assay to identify inhibitors against CDV.

Interestingly, both inhibitors resembled the AS-48 class compounds, which inferred a similar mode of binding and action. Indeed, high resolution structural studies of prefusion MeV F-protein in complex with FIP and AS-48 revealed a common binding pocket located at the junction of the head and stalk domains [26]. Moreover, functional studies suggested that such inhibitors may increase the energy threshold required for F-activation in turn efficiently preventing F-refolding and ensuing membrane fusion activity (reviewed in [49]). F2736-3056 and F2261-0043 may hence bind to the same pocket microdomain of the CDV F prefusion state and block its activity. Supporting this hypothesis, our in silico analyses indicated the possibility that both identified compounds may indeed dock into the similar microdomain in CDV F. Of note, the shallow groove, which extends towards the solvent from the deep hydrophobic pocket, enables accommodation of compounds carrying additional fused groups to the 2-phenylacetanilide scaffold (Figure 5B). Interestingly, F2736-3056 contains a supplementary morpholine moiety, which points outside of the pocket (Figure 5A). This moiety may support an additional hydrogen bond interaction with a lysine residue (Lys581 in CDV F and Lys469 in MeV F) locating in the F-stalk domain, which resembles the mode of interaction of FIP in the MeV F-protein (Figure 5A). This additional interaction may thus further stabilize the prefusion state of the CDV F-protein; an observation which is in good agreement with our functional studies (Figure 4). Indeed, compared to 3G, F2736-3056 preserved substantial fusion-inhibitory activity against a hyperfusogenic, 3G-escape, F-mutant.

Using this assay, we aim to screen additional libraries to discover compounds with novel scaffolds and better potency that would be necessary to increase our arsenal towards the development of multiple antiviral drugs against CDV. Simultaneously, by employing this assay, we also intend to identify compounds that could improve our basic understanding of the molecular mechanisms underlying morbilliviral cell-entry and spread. Furthermore, such molecular tools may enhance the quality of cryo-EM-based prefusion CDV F-structures. In turn, high-resolution CDV F-structures may set the stage to rationally refine the existing fusion inhibitors and/or to act as templates for the design of novel binders that can eventually be utilized synergistically. Indeed, compared to a monotherapy approach, a combined therapy (e.g., targeting multiple players of the viral-induced membrane fusion process) is likely to match with more realistic clinical requirements.

In summary, in this study we present an HTS-compatible, highly-sensitive quantitative cell-based fusion assay that can be employed to further refine our knowledge of the morbilliviral entry and spread mechanisms, as well as to discover molecules able to inhibit such processes.

## Data Availability

The data presented in this study are available in this article and on request from the corresponding author.
